# Stinkopathists

**Published:** 1886-04

**Authors:** 


### "Stinkopathists."

Under this head the *Mississippi Valley Medical Journal* describes a school of medical men, altogether too numerous in every community. The following is their confession of faith or code:

1. When a physician has located in a place, he should advertise himself in every conceivable way; let him practice a year free for some blatherskite newspaper editor to get him to blow for him. He should sign his name with F. R. C. S., M. D.

2. A physician should never, under any circumstances, or for any consideration, allow himself to speak favorably of any other doctor, and if some one else does so in his presence, offset the remark as far as possible by one of his own.

3. If a neighboring physician happens to lose a couple of cases of diphtheria, it is the bounden duty of any struggling stinkopathist to remark in the right place, in a casual way, that he is sorry for Dr. Blank's misfortune, and then add, that since adopting the new treatment, he hasn't lost a case from the disease.

4. If a physician is called accidentally, or through the courtesy of another doctor, to consult in a case of simple remittent fever, he should examine the case a long time, and ask about eleven hundred questions which he knows, from the nature of the case and the good sense of the physician in charge, have not been asked, and when he has ascertained what the treatment has been, it shall be his duty to suggest a change at any rate. If the physician in charge is giving sulphate of quinine, suggest the bromide or valerianate; and he should always make it a point to have these things somewhere about his old clothes, as the patient might recover entirely before the prescription could be sent for.

5. When a physician is called to consult in a case of puerperal fever, for instance, and the patient is not likely to recover under any plan of treatment, and the consulting doctor fails to get an invitation to treat the patient farther in connection with the physician in charge, he should go about town and tell everybody he can that the patient is not dangerously ill at all, and will be up in a few days if properly handled.

6. If a physician is called to consult in a case of cerebro-spinal meningitis, and the patient is in *articulo mortis* when he arrives, he should not examine at all, but take in the situation at a glance, remembering that his opportunity for "turning a Jack" in the case will not last long, and hurry the other doctor out, and stay just long enough to find out what he hasn't done. Then it shall be his duty to pull off his coat, push up his sleeves and go to work. He must order water heated, and a tub full cold from the well or cistern, put some brick in the fire, go into his saddle-bags, if he be a country doctor, and get out something and give it to the patient, get out his hypodermic syringe, but don't use it, give the patient half a pound of epsom salts if he can swallow. Send a fellow five miles for a big old-fashioned syringe, and another for some whisky, and by this time the patient is in paradise. The physician shall then make sure of death, slowly gather up his instruments and things, and sorrowfully bid his audience adieu, and tell some one he may meet as he goes away, that he was too late.

When you find a doctor who adheres religiously to such rules, you have a genuine uncomplicated case of cussedness that is past the art of man to cure, no matter where he hails from, to what school he belongs, how large his practice, or what hold he may have upon the hearts and pockets of the laity. And to talk to him about being honorable is like singing psalms to a deceased horse.

